# Four new non-spiny *Solanum* (Solanaceae) species from South America

**DOI:** 10.3897/phytokeys.44.8693

**Published:** 2015-01-13

**Authors:** Tiina Särkinen, Paúl Gonzáles, Sandra Knapp

**Affiliations:** 1 Royal Botanic Garden Edinburgh, 20A Inverleith Row, EH3 5LR Edinburgh, United Kingdom; 2 Laboratorio de Florística, Departamento de Dicotiledóneas, Museo de Historia Natural - Universidad Nacional Mayor de San Marcos, Avenida Arenales 1256, Apartado Postal 14-0434, Lima, Peru; 3 Department of Life Sciences, Natural History Museum, Cromwell Road, SW7 5BD London, United Kingdom

**Keywords:** Tropical Andes, Solanaceae, Peru, endemism, Morelloid clade, Solanum section Solanum, Potato clade, Solanum section Basarthrum, Andes tropicales, Solanaceae, Perú, endemismo, clado Morelloide, Solanum sección Solanum, clado Potato, Solanum sección Basarthrum

## Abstract

Four new species of “non-spiny” *Solanum* from South America are described. *Solanum longifilamentum* Särkinen & P.Gonzáles, **sp. nov.** (Morelloid clade) is widespread from Ecuador to Bolivia and is most similar to *Solanum macrotonum* Dunal from Central and northern South America. *Solanum antisuyo* Särkinen & S.Knapp, **sp. nov.** (Morelloid clade) is found on the eastern Andean slopes in Ecuador, Peru and Bolivia and is most similar to the widespread lower elevation species *Solanum polytrichostylum* Bitter. *Solanum arenicola* Särkinen & P.Gonzáles, **sp. nov.** (Morelloid clade) is found in low elevation habitats on the eastern Andean slopes and in Amazonia of Peru and Bolivia and is most similar to the higher elevation species *Solanum aloysiifolium* Dunal of Bolivia and Argentina. *Solanum mariae* Särkinen & S.Knapp, **sp. nov.** (Potato clade) is endemic to Cajamarca Department in Peru, and is most similar to the widespread *Solanum caripense* Dunal. Complete descriptions, distributions and preliminary conservation assessments of all new species are given.

## Abstract (es)

Se describen cuatro species nuevas de *Solanum* de América del Sur. *Solanum longifilamentum* Särkinen & P. Gonzáles, **sp. nov.** (clado Morelloide) se encuentra de Ecuador a Bolivia y es muy similar a *Solanum macrotonum* Bitter de Centroamerica y la parte norte de América del Sur. *Solanum antisuyo* Särkinen & S.Knapp, **sp. nov.** (clado Morelloide) se encuentra en las vertientes orientales de los Andes en Ecuador, Peru y Bolivia y es similar a *Solanum polytrichostylum* Bitter, una especie de amplia distribución de elevaciones menores. *Solanum arenicola* Särkinen & P. Gonzáles, **sp. nov.** (clado Morelloide) se encuentra en las vertientes orientales de los Andes y la selva baja de Peru y Bolivia, es muy parecida a una especie que se encuentra a mayor altitud en Bolivia y Argentina, *Solanum aloysiifolium* Dunal. *Solanum mariae* Särkinen & S.Knapp, **sp. nov.** (clado Potato) es endémica del departamento de Cajamarca (Perú), y es similar a *Solanum caripense* Dunal, una especie de amplia distribución en los Andes. Se presenta descripciones completas, mapas de distribuciones y evaluaciones preliminares de conservación de todas las especies descritas aquí.

## Introduction

*Solanum* is one of the largest of flowering plant genera (Frodin 2004) and its centre of diversity is in South America (Knapp 2002). The Andes are a hot-spot for *Solanum* diversity and *Solanum* is one of the most species-rich vascular plant genera in the Andes (Jørgensen et al. 2011). In Peru alone 272 species of *Solanum* occur, of which 83 are currently listed as endemic (Knapp et al. 2006; Särkinen et al., in review). Many new species continue to be described (e.g., Anderson et al. 2006; Stern and Bohs 2010; Knapp 2010a,b; Farrugia and Bohs 2010; Tepe et al. 2012; Särkinen et al. 2013) from the Andean region, discovered both in the field and in the herbarium. *Solanum* comprises 13 major clades of which the spiny solanum clade corresponding to subgenus *Leptostemonum* is the largest (Särkinen et al. 2013). The “non-spiny” solanums are distributed amongst a series of monophyletic groups, some of which have been treated recently (e.g., Knapp 2002, 2013; Tepe et al. 2011) while others are in the process of revision. Traditional sectional names for groups of non-spiny solanums are still in wide use, but many of these groups are very different in circumscription to these traditional concepts. Stern et al. (2011) provided an assessment of the clades of spiny solanums in the New World, but the “non-spiny” solanums have not been similarly treated.

We here describe four new species that came to light on recent field work as part of a project investigating the distributional ranges of Solanaceae in this megadiverse region. Descriptions are based on field work and examination of herbarium specimens from 20 herbaria (BH, BM, COL, CORD, CPUN, DUKE, E, F, GH, GOET, HUSA, HUT, K, MO, MOL, NY, S, UDBC, US, USM). All specimens are cited in the text and full data are provided in the supplemental file and on Solanaceae Source (http://www.solanaceaesource.org).

## Taxonomic treatment

### The Morelloid clade

The Morelloid clade is one of the larger monophyletic groups of “non-spiny” solanums, and contains the type of the genus (*Solanum nigrum* L., a European hexaploid species). The clade comprises five groups, four of which only have a few species (e.g., section *Campanulisolanum* Bitter, Barboza 2005). The largest group of species (ca. 52 species) are those related to *Solanum nigrum* that are often referred to as section *Solanum*. Members of this group occur worldwide with a centre of diversity in the Andes. This large group of species is morphologically relatively homogenous and is distinguished by its herbaceous habit, inflorescences usually positioned along the internodes, small flowers and fruits, and the usual possession of stone cells in the fruits (Bitter 1911). Stone cell aggregates are small, seed-like structures that are usually spherical (rather than flattened as are the seeds) and can most easily be seen in pressed specimens through the fruit wall. Although some studies have been done to clarify the taxonomy of the Old World and North American species (Edmonds 1977, 1978; Schilling 1981), monographic treatment of the entire section is needed to aid species identification and to clarify synonymy for the ca. 580 published names for these taxa. This is especially the case for South America, where most species diversity within the section lies (Edmonds 1972; Barboza et al. 2013), and where many taxa remain to be re-circumscribed and new species described. Recent work towards a taxonomic revision of this complex group has resulted in the description of two new species in this group (Särkinen et al. 2013; Manoko et al. 2013), both of which had previously been subsumed within other widespread taxa. The three species described here were similarly considered as part of poorly understood widespread taxa, but field and herbarium studies have shown them to be distinct.

#### Solanum longifilamentum

Taxon classificationPlantaeSolanalesSolanaceae

Särkinen & P. Gonzáles sp. nov.

urn:lsid:ipni.org:names:77144531-1

Figs 1 –2

##### Diagnosis.

Like *Solanum macrotonum* Bitter, but differing in consistently narrower oblong-lanceolate leaves, few-flowered inflorescences, longer calyx lobes which elongate in fruit, filaments a minimum of half the length of anthers, and less exerted style exceeding to only 1 mm beyond the anther cone.

##### Type.

**Peru. Pasco:** Prov. Oxapampa, Dist. Huancabamba, Parque Nacional Yanachaga-Chemillén, sector Tunqui, riberas del rio Muchuymayo, alrededores del hito PNYC, 1790 m, 22 Oct 2008 (fl, fr), *M. Cueva, A. Peña, R. Rivera & M. Moens 276* (holotype: USM [USM-00268971]; isotypes: HOXA, HUT, MO [acc. 6455431]).

##### Description.

Delicate herb to small subshrub, woody at base, 20–100 cm tall, single stemmed or occasionally branching at the base. Stems 2–4 mm in diameter at the base, terete to ridged, often purple-tinged, sparsely pubescent with appressed 1–2-celled simple uniseriate trichomes ca. 0.2 mm long. Sympodial units difoliate, not geminate. Leaves simple, 2.5–12.0 cm long, 1.0–4.0 cm wide, ovate-lanceolate; adaxial surface glabrous; abaxial surface with appressed 1–2-celled simple uniseriate trichomes like those of the stem along the veins; primary veins 4–8 pairs; base cuneate to attenuate, slightly unequal and oblique; margins entire; apex acuminate; petiole 0.5–1.0 cm long, sparsely pubescent with simple uniseriate trichomes like those of the stems and leaves, especially on young growth. Inflorescences lateral and internodal, 1.5–3.0 cm long, simple, with 3–5(6) flowers often all apparently arising from the same place, sparsely pubescent with simple uniseriate trichomes like those of the stems and leaves; peduncle 1.0–1.5 cm long, often tinged with purple; pedicels 0.5–0.6 cm long, ca. 0.4 mm in diameter at the base and 0.5 mm at apex, straight and spreading at anthesis, articulated at the base; pedicel scars closely spaced a maximum of 1 mm apart. Buds conical, white, occasionally purple-tinged towards the base, the corolla strongly exerted from the calyx tube long before anthesis. Flowers 5-merous, all perfect; calyx tube ca. 1.5–2.0 mm long, the lobes 1.0–1.5 mm long, deltate to traingular with acute apices, slightly reflexed at anthesis, sparsely pubescent with simple uniseriate trichomes like those of the stems and leaves; corolla 5–6 mm in diameter, stellate, whitewith a yellow, purple or black central star at the base, lobed 2/3 to nearly to the base, the lobes ca. 3.0–3.5 mm long, 1.5–2.0 mm wide, strongly reflexed at anthesis, later spreading, purple towards tips, densely pubescent abaxially with 1–2-celled simple uniseriate trichomes, these usually shorter than the trichomes of the stems and leaves; filament tube 1.0–1.2 mm long, pubescent with a few scattered 3–5-celled trichomes at the base adaxially; free portion of the filaments ca. 1.1–1.4 mm long, pubescent like the tube; anthers (1.7-)3.0–3.4 mm long, 0.8–0.9 mm wide, ellipsoid, yellow, poricidal at the tips, the pores lengthening to slits with age; ovary globose, glabrous; style 3.5–4 mm long, exerted only to 0.5–1.0 mm beyond the anther cone, densely pubescent in lower ¼ with 2–3-celled simple uniseriate trichomes; stigma globose, minutely papillate, pale yellow in live plants. Fruit a globose berry, 6–7 mm in diameter, green at maturity or green and turning purplish black when ripe, the surface shiny; fruiting peduncle same as in flower; fruiting pedicels 1.0–1.2 cm long, ca. 0.6 mm in diameter at the base, 0.9 mm at apex, spreading; fruiting calyx lobes 1.8–3.5 mm long, spreading, the tips reflexed. Seeds 35–45 per berry, c. 1.2 mm long, c. 1.1 mm wide, concave-reniform, narrower at one end, brownish orange, the sub-lateral hilum positioned towards the narrower end of the seed, the testal cells pentagonal in outline; stone cells few per fruit.

**Figure 1.**
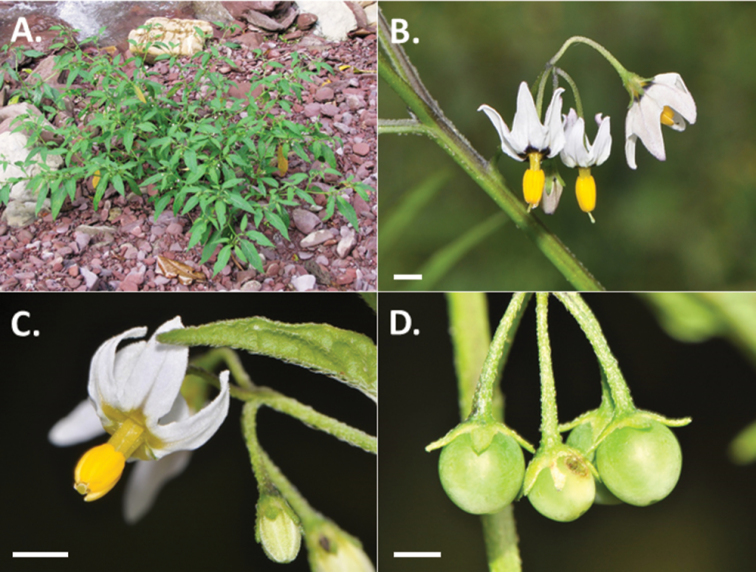
Photos of *Solanum longifilamentum*. **A** Habit (*Cueva et al.* 276) **B** Flowers at full anthesis (*Särkinen et al.* 4030) **C** Buds and flowers, floral type without black central star (*Knapp et al.* 10545) **D** Fruits with spreading calyx lobes (*Knapp et al.* 10545). Scale bars = 2 mm. Photos by S. Knapp (**C, D**), M. Cueva (**A**), and T. Särkinen (**B**).

##### Distribution.

Ecuador, Peru, and Bolivia on the eastern slopes of the Andes growing in mid-elevation montane forests in moist areas, along roadsides, often amongst mosses and small herbs, associated with Ericaceae and Asteraceae shrubs and herbs, Lauraceae, *Alnus acuminata* Kunth, *Cecropia* (Urticaceae), *Clusia* (Clusiaceae), *Fuchsia* (Onagraceae), *Hedyosmum* (Chloranthaceae), *Weinmannia* (Cunoniaceae), *Miconia* (Melastomataceae), and tree ferns; between (800-) 1,000–2,800 (-3,500) m elevation.

**Figure 2.**
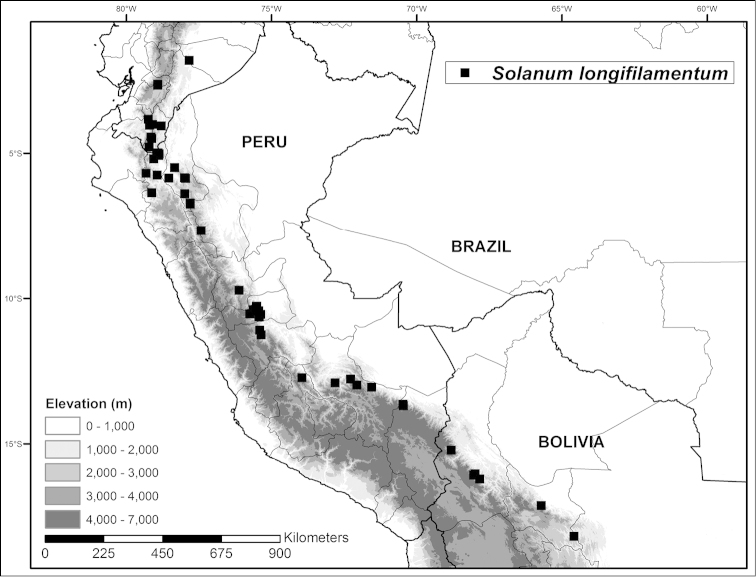
Distribution map of *Solanum longifilamentum* along eastern flanks of Central Andes in southern Ecuador and Peru.

##### Ecology.

Flowering and fruiting throughout the year, with a peak of fruiting in March–July.

##### Common names.

Mortiño (Spanish; *Särkinen et al. 4577*); Wampishkúr (Shuar Jívaro; *Lewis 14172*).

##### Uses.

Stems and leaves crushed and applied with achiote (*Bixa orellana* L., Bixaceae) warm to treat skin irritations (papera) (*Lewis 14172*).

##### Etymology.

The species is named based on its uniquely long filaments in relation to the anthers which distinguish it from the closely related *Solanum macrotonum*.

##### Conservation status.

The preliminary conservation status (IUCN 2010) of *Solanum longifilamentum* is here considered of least concern (LC) based on the relatively large extent of the species occurrence (EOO, c. 781,800 km^2^), although the actual area of occupancy (AOO) is small (228 km^2^) and would merit status as endangered (EN). Many recent collections exist, indicating that populations are not in decline, and as are most members of the Morelloid clade, *Solanum longifilamentum* is a weedy plant of disturbed areas.

##### Specimens examined.

**BOLIVIA. Cochabamba:** Chapare: Along highway from Villa Tunari to Cochabamba, 43 km SW of bridge over Río Espírito Santo, 17°14'42"S, 65°71'17"W, 1320m, 1 May 2007, *M. Nee 55273* (MO); **La Paz:** Bautista Saavedra: Area Natural de Manejo Integrado Apolobamba, carretera entre Charazani y Camata, 15°12'48"S, 68°49'08"W, 1870m, 19 Aug 2008, *A. Fuentes 13261* (NY,USM); Murillo: 44.0 km below Lago Zongo dam, vicinity of Cahua hydroelectric plant, 16°05'00"S, 68°01'67"W, 1200m, 12 Sep 1983, *J.C. Solomon 10806* (MO); Murillo: Valle del Río Zongo, 40.2 km al N (abajo) de la cumbre, 16°04'S, 68°02'W, 1600m, 30 Apr 1990, *J.C. Solomon 18869* (G, MO); Nor Yungas: Carretera 3, 16.2 km SW of Yolosa jct toward Unduavi, 16°13'15"S, 67°49'58"W, 2078m, 12 Nov 1976, *C. Davidson 4976* (MO); **Santa Cruz:** Within the Flora de la Region del Parque Nacional Amboró, but above the 700m contour, 18°06'30"S, 063°57'00"W, 1465m, *M. Nee 37245* (MO); **ECUADOR. Cañar:** Iganilla Pass on Pan America Highway between Cañar and Biblian, 2°37'S, 78°55'W, 3500m, 12 Dec 1980, *L.B. Holm-Nielsen 28998* (BM,MO); Iganilla, S of the pass on Pan American Highway between Cañar and Biblian, 2°37'S, 78°55'W, 3400m, 12 Dec 1980, *L.B. Holm-Nielsen 29039* (BM, MO); Carretera Cañar-Azoguez, desvio a Moloboc-Grande-Molon-Ventana, 1°46'00"S, 78°36'W, 3200–3450m, 14 Aug 1987, *V. Zak 2446* (MO); **Loja:** Reserva Ecológica Tapichalaca (Fundación Jocotoco), between Yangana and Valledolid, 4°29'42"S, 79°07'55"W, 2500m, 3 Apr 2005, *L. Bohs 3408* (NY, QCNE); Rd from Loja to Yangana, 4°12'00"S, 79°10'12"W, 11 Jul 1986, *W.G. D’Arcy 16452A* (MO); 20 kms from Yangana, 4°25'59"S, 79°10'00"W, 2743m, 12 Jul 1986, *W.G. D’Arcy 16476* (MO); Parque Educacional y Recreacional Universidad Nacional de Loja, vicinity of Loja, 4°02'02"S, 79°11'87"W, 2100–2300m, 28 Oct 1994, *S. Knapp 9092* (BM, MO); between Loja and Saraguro, 2316m, 14 Jul 1984, *W.G. D’Arcy 15746* (MO, NY); **Pastaza:** Tsurakú (Pitirishca), km 51 S on rd from Puyo to Macas, 1°51'S, 77°48'W, 800m, 1 Aug 1988, *W.H. Lewis 14172* (MO); **Pichincha:** Along rd from Tandayapa to Nono, 4.1 km S of Tandayapa, 0°00'36"S, 78°39'23"W, 1828m, 4 Sep 2007, *T.B. Croat 98274* (MO); **Zamora-Chinchipe:** Fundación Arco Iris, between Loja and Zamora, trail from field station to Río San Francisco, 3°59'20"S, 79°05'35"W, 2200m, 5 Apr 2005, *L. Bohs et al. 3434* (BM, QCNE); 10 km S of Namirez and Rio Zimora, vicinity of Nambija, along rd to mine headquarters ca. 5 km long, just south of Nambija, 4°03'44"S, 78°47'29"W, 1779m, 23 Jul 2004, *T.B. Croat 92009* (BM); Prov. Chinchipe, region of Guaramizal, Parroquia Zumba, Quebrada Tarrangamí, near cabin of Sandy León, W of Escuela Byron Jiménez, just S of Las Pircas, 4°46'50"S, 79°12'33"W, 2000m, 29 Mar 2005, *L. Bohs et al. 3354* (BM, QCNE, UT); **PERU. Amazonas:** Prov. Bagua, third camp SE of la Peca, 1867–2179m, 10 Oct 1978, *P. Barbour 3958* (USM); Prov. Bongará, outside of main entrance to privately owned bird sanctuary just outside of the Alto Mayo protected area, ca. 2 km E of Area de Conservación Privada Arba Patricia-Alto Nieva, 13 Oct 2010, *F. Farruggia et al. 2753* (BM, HAO, NY, UT); Prov. Chachapoyas, Torrecilla, nr Turbaco, 2300m, 31 Mar 1979, *C.M. Ochoa 13254* (US); Dist. Leymebamba, Bosque Alto Atuen, 6°45'20"S, 77°48'07"W, 2583m, 27 Jun 2009, *R.W. Bussmann et al. 15823* (HUT); Prov. Luya, km 18–20 on rd from Nuevo Tingo to Kuelap, just before bridge at Choctamal, 6°23'13"S, 77°59'10"W, 2610m, 19 Apr 2013, *T. Särkinen et al. 4611* (USM); **Ayacucho:** Prov. La Mar, Aina, between Huanta and Río Apurimac, 12°43'40"S, 73°57'07"W, 1883m, 7 May 1929, *E.P. Killip & A.C. Smith 23116* (US); Dist. Anco, alrededores de Buena Gana, ca. 8.5 km lineales al WNW de San Antonio, 1775m, 21 Apr 2007, *J. Roque 5484* (USM); **Cajamarca:** Prov. Chota, a 1 km de Paraguay (Queroto-La Granja), 2250m, 7 Aug 1994, *S. Leiva et al. 1390* (F); Dist. Querocoto, Hacienda La Granja, quebrada San Lorenzo, 6°22'34"S, 73°08'18"W, 2100–2500m, 2 Dec 2012, *P. Gonzáles et al. 2108* (USM); Prov. Jaén, Sallique, camino entre El Espino y Tablón, 5°41'S, 79°19'W, 2500m, 25 Jul 1998, *J.Campos et al. 5379* (MO, USM); Prov. San Ignacio, San José de Lourdes, Santo Tomás, 5°01'S, 78°52'W, 1900m, 7 Apr 1997, *J. Campos & S. Corrales 3796* (BM, F, MO, USM); San José de Lourdes, localidad Laurel, 5°01'S, 78°56'W, 1500–1600m, 17 May 1997, *J. Campos & W. Vargas 3908* (MO, MOL, USM); San José de Lourdes, 5°45'S, 78°56'W, 1020m, 18 Feb 2000, *J. Campos & R. Vásquez 6472* (MO, MOL, NY, USM); Dist. Huarango, Nuevo Mundo, Caseiro Pisaguas, a 2 horas del poblado y al N, margen derecha quebrada Santa Rosa, 5°51'08"S, 78°32'12"W, 1700m, 11 Nov 1997, *E. Rodríguez R. 1909* (BM, HUT, MO); Dist. San Ignacio, El Chaupe, 5°11'12"S, 79°03'12"W, 1880m, 10 Oct 2010, *F. Farruggia et al. 2720* (BM, HAO, NY, UT); San José de Lourdes, campamento Zural, camino al cerro Picorana, 4°59'15"S, 78°54'03"W, 2010m, 29 Jan 1999, *C.Díaz et al. 10555* (MO, MOL, USM); Tabaconas, Santuario Nacional Tabaconas-Namballe, Quebrada Chichilapa grande, 2190m, 11 Nov 1998, *C. Díaz 9972* (MO,NY,USM); Along mud track between San Jose de Lourdes and Monterey de le Frontera, past the village of Diamantes, 4°59'03"S, 78°55'41"W, 1587m, 16 Apr 2013, *T. Särkinen et al. 4577* (USM); **Cusco:** Prov. Calca, ca. 2 km up Lares branch of Calca-Quellouno rd (from junction of the Amparaes branch and the Lares branch), just above large bridge nr rd junction on way to Lares, 12°58'27"S, 72°03'32"W, 2589m, 16 Mar 2012, *S. Knapp et al. 10454* (BM, USM); Prov. La Convención, Dist. Ocobamba, Versalles, 12°46'37"S, 72°16'58"W, 1853m, 21 Nov 2007, *L. Valenzuela et al. 10353* (BM); Prov. Paucartambo, entre los km 128–131 de la carretera Paucartambo-Pilcopata, 2350–2400m, 4 Jul 1992, *A. Cano & O. Riofrío 5402* (USM); Entre los km 126–128 de la carretera Paucartambo-Pilcopata, 2500–2600m, 5 Jul 1992, *A. Cano & O. Riofrío 5516* (USM); Entre los km 128–131 de la carretera Paucartambo-Pilcopata, 2350–2400m, 4 Jul 1992, *A. Cano & O. Riofrío 5455* (USM); 13°03’ S, 71°33’ W, 1500m, Aug 2010, *P. Chambi & J. Chambi 21* (USM); **Huánuco:** Prov. Chinchao, San Pedro de Carpish, arriba del tunel, camino a la torre eletrica chica, 2770–2820m, 22 May 2002, *I. Salinas 336* (USM); Dept. Chinchao, San Pedro de Carpish, arriba del tunel, 2770–2820m, 3 May 2005, *I. Salinas & H. Beltrán 1144* (USM); **Junín:** Prov. Chanchamayo, on rd from San Ramon to Puyusacha, a private conservation area, 11°05'31"S, 75°24'25"W, 949m, 30 May 2013, *T. Särkinen et al. 4820* (USM); Rd from San Ramón past Vitoc to Union Mantish, past Rio Chilpes, 11°15'05"S, 75°21'22"W, 1309m, 2 Jun 2013, *T. Särkinen et al. 4834* (USM); **Pasco:** Prov. Oxapampa, 2–4 km N of Mallampampa, 10°32'S, 75°45’ W, 2200–2400m, 22 Jan 1984, *D.N. Smith & J. Canne 5857* (MO,USM); Parque Nacional Yanachaga-Chemillén, sector San Daniel, zona de amortiguamiento, 10°25'36"S, 75°26'35"W, 2387m, 28 Dec 2008, *M. Cueva & A. Peña 418* (HUSA,USM); Parque Nacional Yanachaga-Chemillén, sector San Tunqui, camino hacia Maria Puñis, 10°16'19"S, 75°30'35"W, 1895m, 6 Feb 2009, *M. Cueva & A. Peña 444* (HUSA); Dist. Huancabamba, Zona de amortiguamiento del Parque Nacional Yanachaga-Chemillén, Rio Chillcatambo, parte media, 10°17'28"S, 75°30'32"W, 1852m, 18 Jul 2008, *A. Monteagudo et al. 16710* (USM); Dist. Huancabamba, Parque Nacional Yanachaga-Chemillén, parte alta de la trocha Tunqui-Cajonpata, sector Tunqui, 10°16'15"S, 75°30'23"W, 1950m, 31 Oct 2007, *A. Monteagudo et al. 15798* (USM); at Ulcumano Ecolodge nr Oxapampa, 10°38'11"S, 75°26'05"W, 2239m, 9 Jun 2013, *T. Särkinen et al. 4856* (USM); Dist. Huancabamba, Sector Tunqui, parte media de la quebrada Muchuy Mayo, 10°17'30"S, 75°31'05"W, 1800m, 29 Oct 2007, *A. Monteagudo et al. 15724* (BM, USM); Dist. Oxapampa, parte media de la quebrada San Alberto (zona de amortiguamiento), 10°33'00"S, 75°22'39"W, 2135m, 8 May 2007, *A. Monteagudo et al. 13936* (BM); Dist. Huancabamba, carretera San Daniel-Tunqui, 10°23'06"S, 73°32'54"W, 1645m, 25 May 2009, *R. Vásquez et al. 35721* (USM); Dist. Huancabamba, sector Tunqui, Parque Nacional Yanachaga-Chemillén, camino hacia Maria Punis, 10°16'31"S, 75°30'59"W, 1895m, 18 Oct 2008, *M. Cueva et al. 216* (USM); Dist. Huancabamba, sector Tunqui, 10°16'42"S, 75°31'01"W, 1784m, 11 Nov 2008, *J.R. Ayerbe & D. Heredia 194* (USM); Dist. Huancabamba, sector San Daniel, Parque Nacional Yanachaga-Chemillén, 10°26'27"S, 75°26'30"W, 2240m, 24 Feb 2009, *M. Cueva & R. Rivera 474* (USM); **Puno:** Prov. Carabaya, Ollachea to San Gaban, 1000–2000m, 17 Jul 1978, *M. Dillon et al. 1115* (MO); Prov. Carabaya, km 258–255 on rd to Juliaca from San Gaban, just before Charcaneque, 13°38'30"S, 70°28'12"W, 1550m, 19 Mar 2012, *T. Särkinen et al. 4029* (USM); Km 250 on rd to Juliaca from San Gaban, just before Uruhuasi, 13°41'23"S, 70°27'26"W, 1918m, 19 Mar 2012, *T. Särkinen et al. 4030* (BM, USM); Prov. Sandia, 2100–2200m, 14 May 1966, *R.Ferreyra 16721* (USM); **San Martín:** Prov. Mariscal Cáceres, between the la playa and Las Papayas camps, 7°00'S, 77°00'W, 2700m, 18 Aug 1986, *K. Young 4265* (USM).

##### Discussion.

*Solanum longifilamentum* is most similar to *Solanum macrotonum* Bitter of Central and northern South America but these species can be distinguished based on calyx lobes (size and shape) in flower and fruit, anther: filament length ratio, and the length of the style. *Solanum longifilamentum* has oblong, calyx lobes 1.0–1.5 mm long that are slightly spreading in fruit, while *Solanum macrotonum* has smaller, 0.5–1.0 mm long, triangular lobes that are tightly appressed to the mature fruit. Filaments are a minimum of half the length of anthers in *Solanum longifilamentum* compared to *Solanum macrotonum* where filaments are always clearly smaller in relation to anthers. Styles are exerted to only 0.5–1.0 mm beyond the anther cone in *Solanum longifilamentum*, but extend 1.5–3.5 mm beyond the anthers in *Solanum macrotonum*. Although leaf shape is generally variable within most *Solanum* species, *Solanum longifilamentum* has consistently narrower, oblong-lanceolate leaves compared to the more ovate leaves of *Solanum macrotonum*. Other members of the Morelloid clade with which *Solanum longifilamentum* could be confused include *Solanum americanum* Mill. and *Solanum pseudoamericanum* Särkinen, P. Gonzáles & S.Knapp both of which have smaller anthers c. 1.0–1.5 mm long and curved or included styles, and *Solanum zahlbruckneri* Bitter that is a larger, broadly spreading shrub up to 2 m high, with larger, violet corollas up to 2 cm in diameter.

Some infraspecific variation in anther size and pubescence can be noted within *Solanum longifilamentum*. Some specimens from throughout the species range have shorter and slightly narrower anthers 1.7-2.0 mm long and 0.6-0.7 mm wide (*Smith & Canne 5857; Salinas 1144; Knapp et al. 10454; Campos 5379; Cueva & Rivera 474; Gonzáles et al. 2108*). These specimens also have denser leaf pubescence on both surfaces, especially on young growth, and more broadly ovate leaves. The variation appears to correlate with environmental conditions rather than geography, where with denser pubescence and smaller anthers seem to grow in drier habitats based on herbarium specimen labels plants.

Specimens belonging to this new taxon have been identified under various names, including *Solanum macrotonum* and *Solanum nigrescens* Martens & Galeotti, neither of which occur in Peru.

#### Solanum antisuyo

Taxon classificationPlantaeSolanalesSolanaceae

Särkinen & S.Knapp sp. nov.

urn:lsid:ipni.org:names:77144534-1

Figs 3 –4

##### Diagnosis.

Like *Solanum polytrichostylum* Bitter, but differing in having either simple or once-branched inflorescences with pedicels spaced c. 1–3 mm apart along the rachis, more reduced, minute calyx lobes, ellipsoid rather than spherical fruits, and larger brown-coloured seeds.

##### Type.

**Peru. Cusco:** Prov. Paucartambo, 1 km from Puesto de Vigilancia of Parque Nacional de Manu on rd from Paucartambo to Pilcopata coming from Puesto, 13°12'05"S, 71°37'21"W, 3480 m, 15 Mar 2012 (fl,fr), *S. Knapp, P. Gonzáles, A. Matthews & T. Särkinen 10435* (holotype: USM; isotypes: BM [BM001114929], F, HUSA, HUT, MO).

##### Description.

Stout herb to a shrub up to 1.5 m tall, much branching at base, the individual branches up to 1m long. Stems 2-ridged or slightly winged especially towards base, 0.4–0.6 cm in diameter, purple-coloured especially at leaf nodes, nearly glabrous, sparsely pubescent with simple uniseriate, much reduced 1–3-celled trichomes especially on the often purple coloured young growth. Sympodial units difoliate, not geminate. Leaves simple, 2–17 cm long, 1.2–8.4 cm wide, broadly ovate-lanceolate, membranous to somewhat fleshy; adaxial and abaxial surfaces sparsely pubescent with more or less appressed 1–3-celled simple uniseriate trichomes 0.1–0.2 mm long; primary veins 7–10 pairs; base rounded, decurrent on the petiole; margins entire, often purple tinged; apex acute to acuminate; petiole 0.3–1.2 cm long, occasionally narrowly winged, sparsely pubescent with simple uniseriate trichomes like those of the stems and leaves. Inflorescences 1.4–4.0 cm long, lateral and internodal, simple or once-branched, with 5–14 flowers arising very close together, sparsely pubescent with appressed 1–2-celled simple uniseriate trichomes similar to those on stem and leaves; peduncle 1.0–3.3 cm long, if the inflorescence branched then the peduncle rachis 0.2–0.4 cm long, short and congested; pedicels 1.0–1.2 cm long, 0.5–0.6 mm in diameter at the base tapering gradually to 1.0–1.2 mm in diameter at apex, straight and spreading at anthesis, recurving and becoming woody in fruit, not dehiscing; pedicel scars spaced 0–2 mm apart. Buds conical-ellipsoid, cream-coloured, the corolla strongly exerted from the calyx tube before anthesis. Flowers 5-merous, all perfect; calyx tube 1.5–2.0 mm long, green, the lobes 0.7–0.9 mm long, broadly deltate with rounded apices, purple coloured, sparsely pubescent with 1-celled simple uniseriate trichomes; corolla 12–24 mm in diameter, stellate, white or rarely lilac with a yellow to yellow-green central star at the base, lobed slightly less than halfway to the base, the lobes ca. 9–15 mm long, 4–5 mm wide, spreading to reflexed at anthesis, pubescent abaxially with 1–3-celled simple uniseriate trichomes shorter than the trichomes of the stems and leaves, sparsely pubescent adaxially at base near the filaments with 5–7-celled simple uniseriate trichomes; filament tube ca. 2 mm long, adaxially pubescent with 5–7-celled simple uniseriate trichomes; free portion of the filaments ca. 2 mm long, sometimes slightly longer in two lowermost anthers at anthesis (elongating after anthesis?), pubescent like the tube; anthers ca. (2.8)3.0–3.4 mm long, 1 mm wide, ellipsoid, yellow, poricidal at the tips, the pores lengthening to slits with age; ovary cylindrical, pubescent 2/3 from the base with 2–3-celled simple uniseriate trichomes; style 6 mm long, exerted (0.5)1–2 mm beyond the anther cone, densely pubescent up to 2/3 of the length with 2–3-celled simple uniseriate trichomes at the base; stigma globose, minutely papillate, pale yellow in live plants. Fruit an ellipsoid berry, 8–11 mm in diameter, green turning translucent green-orange when ripe (purple-black in *Knapp et al. 10404* but these affected by pathogens?), the surface smooth and shiny when young, with relatively thick pericarp ca. 0.1 mm; fruiting peduncle woody; fruiting pedicels 11–22 mm long, purple coloured, ca. 1 mm in diameter at the base and 1.5 mm at apex, reflexed and woody in fruit, remaining on the plant after fruit drops; fruiting calyx lobes tightly appressed to the berry, purple-coloured, calyx often splitting into two larger lobes. Seeds 35–45 per berry, ca. 1.1 mm long, ca. 1.7 mm wide, concave-reniform, narrower at one end, brown, the hilum positioned sub-laterally towards the narrower end, the testal cells pentagonal in outline; stone cells few per fruit.

**Figure 3.**
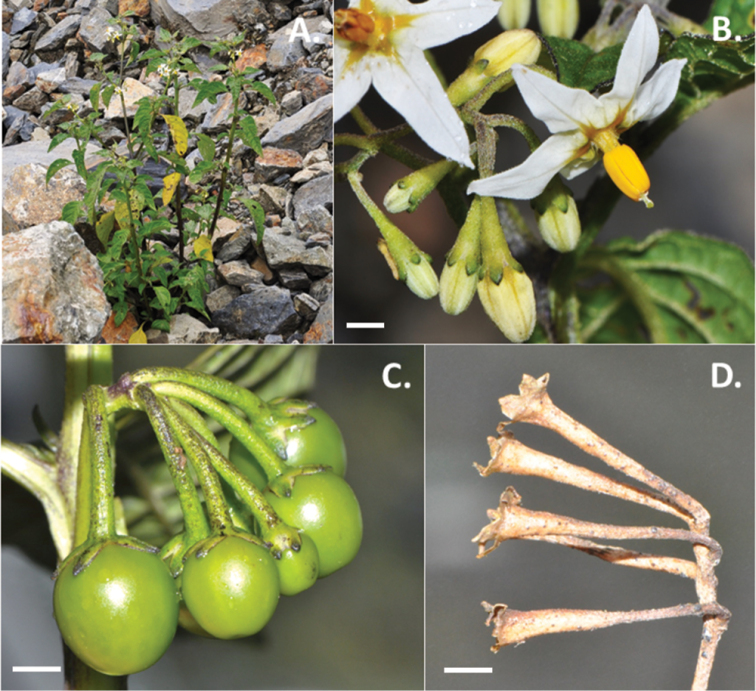
Photos of *Solanum antisuyo*. **A** Buds and flowers, showing the distinct calyx with long tube and minute but thick purple coloured lobes (*Knapp et al.* 10399) **B** Habit, growing in a rocky land slide in gravel (*Knapp et al.* 10399) **C** Woody pedicels of the infrutescence, distinct character in herbarium specimens (*Knapp et al.* 10401) **D** Ellipsoid fruits with reflexed pedicels, and the characteristic appressed calyx lobes that split into two in fruit (*Knapp et al.* 10435). Scale bars = 2 mm. All photos by S. Knapp.

##### Distribution.

Andean Ecuador, Peru, and Bolivia; growing in secondary vegetation, disturbed roadsides, landslides, and gravely slopes in ceja de Selva, montane cloud forest and *Polylepis* forests, associated with *Chusquea* (Poaceae). *Gunnera* (Gunneraceae), *Cecropia* (Urticaceae) and *Weinmannia* (Cunoniaceae); 2,000–3,600 (-3,900) m in elevation.

**Figure 4.**
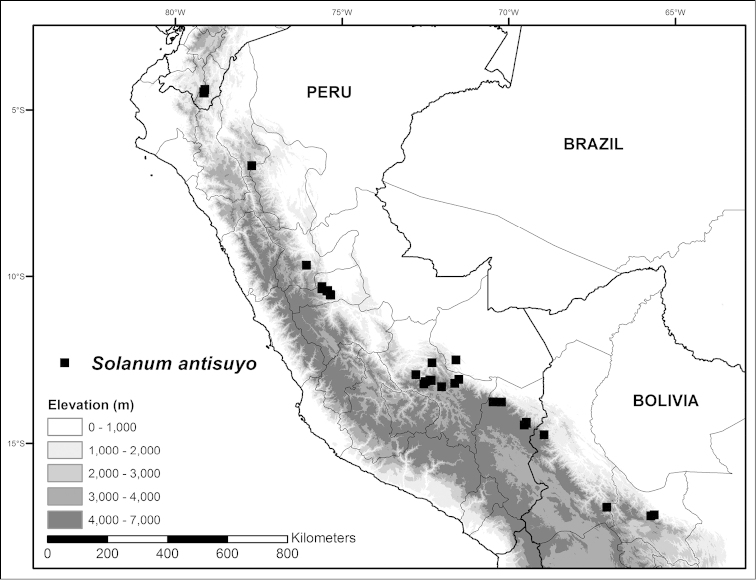
Distribution map of *Solanum antisuyo*, endemic species to Peru.

##### Ecology.

Flowering and fruiting throughout the year, peak in March and June–July.

##### Etymology.

The species name refers to Antisuyo (also Antisuyu), Quechua, for the eastern (*anti*) region (*suyo/suyu*) of the Inca territory, that referred to the lands on the eastern Andean slopes. The species is most diverse along the eastern flanks of the Andes in southern Peru, and the name is chosen to reflect this.

##### Conservation status.

The preliminary conservation status (IUCN 2010) of *Solanum antisuyo* is here considered of least concern (LC) based on the relatively large EOO (ca. 692,500 km^2^), although the AOO (136 km^2^) would merit listing as endangered (EN). The species grows readily in disturbed sites and combined with its wide range, it appears to have relatively low threat status despite the generally increasing human pressure and habitat destruction in the Andes.

##### Specimens examined.

**BOLIVIA. Cochabamba:** Chapare: along highway from Villa Tunari to Cochabamba, at Rio San Jacinto bridge, 54.5 km SW of bridge over Rio Espirito Santo, 17°11'09"S, 65°44'49"W, 1915m, 1 May 2007, *M. Nee 55288* (NY); Chapare: New highway to the Chapare, ca. 4 km beyond Laguna Corani, 3140m, 15 Jul 1994, *N. Ritter 1231* (NY); Chapare: El Limbo, 17°09'38"S, 65°38'22"W, 2900m, 18 Dec 2007, *J. Terán 1757* (NY); **La Paz:** Franz Tamayo: Madidi, Tanhuara Area Natural de Manejo Integrado Apolobamba, bajando del campamento Tanhuara cruzando el Río Pelechuco, 14°44'47"S, 68°56'38"W, 2291m, 11 Jul 2009, *I. Loza 1086* (NY); Inquisivi: Following the slopes E of Communidad Micayani to the Río Khokhoni more or less to the junction with a fork flowing down from Communidad Yamora, and following the Río Khokhoni upstream 1 km from this point, ca. 4 km SE from Inquisivi, 16°91'67"S, 67°10'00"W, 14 Jan 1989, *M. Lewis 35076* (G,MO); Nor Yungas: Unduavi, 3200m, Feb 1914, *O. Buchtien 463* (BM, G, US). **ECUADOR. Loja:** Loja: Parroquia Yangana, rd descending from Cerro Toledo antennas toward Yangana, 4°23'02"S, 79°06'58"W, 3050m, 27 Mar 2005, *L. Bohs 3321* (BM, LOJA, NY, QCNE, UT); Loja: Rd from Yangana to boundary of Zamora-Chinchipe, 2560m, 13 Jul 1984, *W.G. D’Arcy 15728* (NY); **Napo:** Napo-Pastasa, E.N.E of Cayambe Mountain, Oriente trail, 3261m, 6 Dec 1961, *P.C.D. Cazalet 5523* (K); between Tena and Papallacta, 12 Jan 1981, *W.G. D’Arcy 14108* (NY); **Pichincha:** Quito: Termas de Papallacta Hot Springs, 67 km E of Quito nr the Hwy, 0°20'S, 78°10'W, 3300m, 18 Aug 2005, *J.L. Clark 9444* (BM, NY, QCNE, US); Quito: Parroquia, Nono, Reserva Yanacocha, Fundacion de Conservacion Jocotoco, 0°07'00"S, 78°35'10"W, 3500m, 13 May 2007, *J.L. Clark 9534* (NY); **Zamora-Chinchipe:** Rd from Yangana to Zumba, 2438m, 13 Jul 1984, *W.G. D’Arcy 15734* (NY); Rd from Vilcabamba to Valladolid, 27 km from Yangana, 2682m, 12 Jul 1986, *W.G. D’Arcy 16468* (BM,NY); Parque Nacional Podocarpus, km 26 on rd Yangana-Valladolid, 4°29'S, 79°09'W, 2550m, 2 Dec 1988, *J.E. Madsen 75741* (BM). **PERU. Amazonas:** Prov. Chachapoyas, Dist. Leymebamba, Cordillera Yasgolga, slopes W of summit El Rayo, 6°40'01"S, 77°43'05"W, 3287m, 24 Jun 2009, *R.W. Bussmann et al. 15721* (HUT); Prov. Bagua, SE of la Peca, 2362–2461m, 16 Oct 1978, *P. Barbour 4088* (USM); **Cusco:** Prov. La Convención, Dist. Echarate, Quebrada Lorohuachana, sector Laco, Santuario Nacional Megantoni, 3400m, 21 Jun 2008, *L. Hernani A 1037* (HUSA); Dist. Echarate, Quebrada Lorohuachana, sector Laco, Santuario Nacional Megantoni, 3533m, 18 Jun 2008, *L. Hernani A 968* (HUSA); Dist. Echarate, Quebrada Lorohuachana, sector Laco, Santuario Nacional Megantoni, 3400m, 21 Jun 2008, *L. Hernani A 1033* (HUSA); Bosque del Chuyapi, 12°56'33"S, 72°47'04"W, 2100m, 19 Jul 2006, *L. Valenzuela et al. 7282* (USM); Bosque del Chuyapi, 12°56'33"S, 72°47'04"W, 2100m, 19 Jul 2006, *L. Valenzuela et al. 7284* (USM); Ca. 51 km from Ollantaytambo on rd over Abra Malaga to Quillabamba and Quelluno, ca. 10 km below Abra Malaga, Amazon slope, 13°07'15"S, 72°19'34"W, 3877m, 13 Mar 2012, *S. Knapp et al. 10399* (BM, USM); Ca. 57 km from Ollantaytambo on rd over Abra Malaga to Quillabamba and Quelluno, ca. 16 km below Abra Malaga, Amazon slope, 13°06'30"S, 72°21'04"W, 3579m, 13 Mar 2012, *S. Knapp et al. 10401* (BM, USM); Ca. 61 km from Ollantaytambo on rd over Abra Malaga to Quillabamba and Quelluno, ca. 21 km below Abra Malaga, Amazon slope, 13°06'15"S, 72°22'03"W, 3448m, 13 Mar 2012, *S. Knapp et al. 10404* (BM, USM); Ca. 61 km from Ollantaytambo on rd over Abra Malaga to Quillabamba and Quelluno, ca. 21 km below Abra Malaga, Amazon slope, 13°06'15"S, 72°22'03"W, 3448m, 13 Mar 2012, *S. Knapp et al. 10406* (BM, USM); Prov. Paucartambo, 3500–3600m, 14 Jul 1990, *A. Cano 3636* (USM); Parque Nacional de Manu, 3500–3600m, 11 Jul 1990, *A. Cano 4327* (USM); Parque Nacional de Manu, 3600–3700m, 6 Mar 1991, *A. Cano 4593* (USM); Prov. Urubamba, Aguas Calientes, Quebrada Alccamayo, 13°09'01"S, 72°30'17"W, 2050–2200m, 29 Aug 2002, *I. Huamantupa & G. Calatayud 2258* (MO, USM); **Huánuco:** Prov. Huánuco, along rd between Huánuco and Tingo María, 1.1 km N of Carpish Tunnel, ca. km 455, 9°40'S, 76°04'W, 2680m, 1 Jun 1998, *T.B.Croat & M. Sizemore 81568* (BM, MO, USM); Carpish, above Acomayo, lower ceja, 2800m, 17 Jul 1964, *P.C. Hutchison et al. 5931* (MO); Carpish, carretera Huanuco-Tingo Maria, 2700–2900m, 3 Oct 1950, *R. Ferreyra 8164* (MOL, USM); North of Carpish Pass, rd from Huánuco to Tingo Maria, 50–52 km NE of Huánuco, 2350–2430m, 6 Dec 1981, *T. Plowman & P.M. Rury 11141* (HB,USM); Prov. Pachitea, 2450m, 24 Dec 1979, *HHV 001519* (USM); Prov. Tingo Maria, 2900m, 21 Jun 1980, *HHV 3050* (USM); **Junín:** Prov. Huancayo, 3200m, 17 Jun 1972, *D. Tovar T. 5* (USM); **Pasco:** Prov. Oxapampa, Dist. Oxapampa, Parque Nacional Yanachaga-Chemillén, sector San Alberto, alrededores del Refugio el Cedro, 10°32'26"S, 75°21'18"W, 2483m, 26 Apr 2009, *M. Cueva & R. Rivera 625* (HUSA, USM); Dist. Huancabamba, Parque Nacional Yanachaga-Chemillén, sector San Daniel, 10°26'09"S, 75°27'07"W, 2192m, 29 Feb 2008, *R. Vásquez et al. 33798* (USM); Parque Nacional Yanachaga-Chemillén, quebrada diablo fuerte, trocha hacia la parcela Oso-Playa, 10°18'14"S, 75°36'14"W, 2398–2500m, 23 Jun 2008, *A. Monteagudo et al. 16477* (USM); Dist. Huancabamba, Zona de amortiquamiento del P.N. Yanachaga-Chemillén, sector Milpo, 10°22'20"S, 75°36'29"W, 2800m, 23 Sep 2004, *A.Monteagudo et al. 7326* (K); Dist. Huancabamba, Parque Nacional Yanachaga-Chemillén, sector San Daniel, 10°25'48"S, 75°27'00"W, 2500–3500m, 1 Mar 2009, *M. Cueva et al. 492* (USM); **Puno:** Prov. Carabaya, margen derecha del rio que pasa frente al campamento Chacayage, 13°45'48"S, 70°13'07"W, 2600m, 10 Mar 2004, *S. Vilca C. et al. 71* (HUSA); km 238–239 on rd to Juliaca from San Gaban, ca. 8–9 km before Ollachea and Puente Chillichaca, 13°45'30"S, 70°28'09"W, 2451m, 19 Mar 2012, *T. Särkinen et al. 4035* (USM); Prov. Sandia, 8 km from Sandia on rd to Cuyocuyo, 14°22'08"S, 69°28'23"W, 2709m, 21 Mar 2012, *T. Särkinen et al. 4048* (BM, USM); 16 km from Sandia on rd to Cuyocuyo, 14°24'06"S, 69°28'25"W, 2990m, 21 Mar 2012, *T. Särkinen et al. 4049* (BM, USM); in Cuyocuyo outside of a house on main rd to Sandia, 14°27'09"S, 69°32'03"W, 3364m, 21 Mar 2012, *T. Särkinen et al. 4053* (BM, USM).

##### Discussion.

*Solanum antisuyo* is morphologically most similar to *Solanum polytrichostylum* Bitter with which it has been conflated in the past. It can be distinguished by simple or once-branched inflorescences where pedicels are spaced ca. 1–3 mm apart along the short rachis compared to consistently branched inflorescences with the flowers congested at the branch tips in *Solanum polytrichostylum*, and ellipsoid fruits as compared to the spherical fruits of *Solanum polytrichostylum*. *Solanum antisuyo* has a longer calyx tube with more reduced, poorly developed purple-tinged calyx lobes compared to the shorter calyx tubes with slightly larger, triangular calyx lobes in *Solanum polytrichostylum*, and larger brown coloured seeds compared to smaller yellow seeds in *Solanum polytrichostylum*. Furthermore, styles are always more exerted (2–4 mm versus 1–2 mm beyond the anther cone) in *Solanum polytrichostylum*. The two species are also ecologically somewhat distinct, with *Solanum polytrichostylum* restricted to streams and moist road sides, and *Solanum antisuyo* is found in drier areas in gravel, disturbed areas, and landslides. Other members of the Morelloid clade in Peru without glandular trichomes with which *Solanum antisuyo* could be confused include *Solanum probolospermum* Bitter that has smaller, spherical fruits, larger violet corollas that are more rotate in outline, and denser indumentum with longer 3–7-celled simple hairs, and *Solanum pallidum* Rusby (incl. *Solanum planifurcum* Bitter) that is similar to *Solanum probolospermum* but has branched rather than simple hairs.

Variation in growth form and flower colour can be observed in the field, where individuals growing in more humid conditions grow into stout herbs to ca. 1.5 m tall, whilst individuals in drier, higher elevation habitats in rocky landslides are stunted herbs reaching only ca. 40 cm in height. Colour variation in corolla is common within Morelloids and *Solanum* species in general: most specimens of *Solanum antisuyo* have creamy white petals, but occasional specimens with lilac corollas are known (e.g., *Särkinen et al. 4048, 4049*, and *4053*).

#### Solanum arenicola

Taxon classificationPlantaeSolanalesSolanaceae

Särkinen & P. Gonzáles sp. nov.

urn:lsid:ipni.org:names:77144535-1

Figs 5 –6

##### Diagnosis.

Like *Solanum aloysiifolium* Dunal, but differing in having simple, sub-umbellate inflorescences, and a dense indumentum of multicellular glandular-tipped trichomes; also similar to *Solanum subtusviolaceum* Bitter, but differing in having internodal inflorescences, much reduced calyx lobes to only 0.5 mm long, corolla deeply lobed to 2/3 of the way to the base, and a more exerted style extending 2–3 mm beyond the anther cone at anthesis.

##### Type.

**Peru. Madre de Dios:** Prov. Tambopata, in the boat harbor of Infierno, c. 20 km SW by road from Puerto Maldonado, 12°44'06"S, 69°13'47"W, 186 m, 3 Aug 2014 (fl,fr), *T. Särkinen & A. Balarezo 4866* (holotype: USM; isotypes (to be distributed): BM, E, F, GHMDD, HOXA, MO, MOL).

##### Description.

Herb or vigorous, weak-stemmed shrub 0.2–1.5 m tall. Stems angled, sparsely to densely pubescent with simple, translucent, uniseriate 3–8-celled trichomes 0.8–2 mm long with glandular tips; new growth densely pubescent with spreading glandular trichomes like those of the stem. Sympodial units difoliate, not geminate. Leaves simple, 2.6–13 cm long, 0.8–5 cm wide, ovate to broadly ovate, membranous; adaxial surface glabrous; abaxial surface paler or tinged with purple, sparsely pubescent with simple uniseriate trichomes like those of the stem restricted to the veins; primary veins 5–7 pairs; base acute to cuneate and decurrent on the petiole; margins variable in shape from entire to undulate to shallowly lobed; apex acute-acuminate; petiole 0.5–5.0 cm long, sparsely to densely pubescent with glandular trichomes like those of the stems. Inflorescences 2.0–3.5 cm long, lateral and internodal, simple, with 3–8(9) flowers, sparsely to densely pubescent with spreading glandular trichomes like those of the stem; peduncle 1.0–2.4 cm long; pedicels 0.5–0.7 cm long, ca. 0.3 mm in diameter at the base and 0.4 mm at apex, straight and spreading, articulated at the base; pedicel scars unevenly spaced 1.0–2.5 mm apart. Buds ellipsoid, the corolla strongly exerted from the calyx tube long before anthesis. Flowers 5-merous, all perfect; calyx tube ca. 1 mm long, shallow, the lobes 0.2–0.5 mm long, triangular with acute apices, sparsely to densely pubescent with glandular trichomes like those of the stem; corolla 8–12 mm in diameter, stellate, white with a purple-yellow or yellow-green central eye at the base, lobed 2/3 to the base, the lobes ca. 3.5–4.0 mm long, 1.0–1.5 mm wide, strongly reflexed at anthesis, later spreading, densely pubescent abaxially with glandular trichomes like those of the stems, glabrous adaxially; filament tube 1.0–1.2 mm long; free portion of the filaments slightly unequal in length, the lower two ca. 1.5 mm long, the upper three ca. 1.0–1.2 mm long, sparsely pubescent with simple uniseriate 1–3-celled trichomes on the side facing the ovary; anthers 3.0–4.0 mm long, 0.8–0.9 mm wide at base and 0.5–0.6 mm wide at apex, cylindrical, narrowing towards the apex, yellow, poricidal at the tips, the pores lengthening to slits with age; ovary ellipsoid, glabrous; style 4–5.7 mm long, exerted 2.0–3.0 mm beyond the anther cone, densely pubescent up to 2/3 of the length with 1–6-celled simple uniseriate trichomes, these longer at the base and becoming gradually shorter towards the middle; stigma clavate, minutely papillate. Fruit a globose berry, 3.5–7.0 mm in diameter, green, turning black when ripe; fruiting peduncle 2.0 cm long; fruiting pedicels 1.0–2.0 cm long, ca. 0.5 mm in diameter at the base and 0.6 mm at apex, strongly recurved; fruiting calyx lobes appressed to the berry, the tips not reflexed. Seeds 35–45 per berry, ca. 0.8 mm long, ca. 0.6 mm wide, flattened-reniform, narrowing towards one end, yellow, the sub-laterally positioned hilum positioned towards the narrower end, the testal cells pentagonal in outline; stone cells few per fruit.

**Figure 5.**
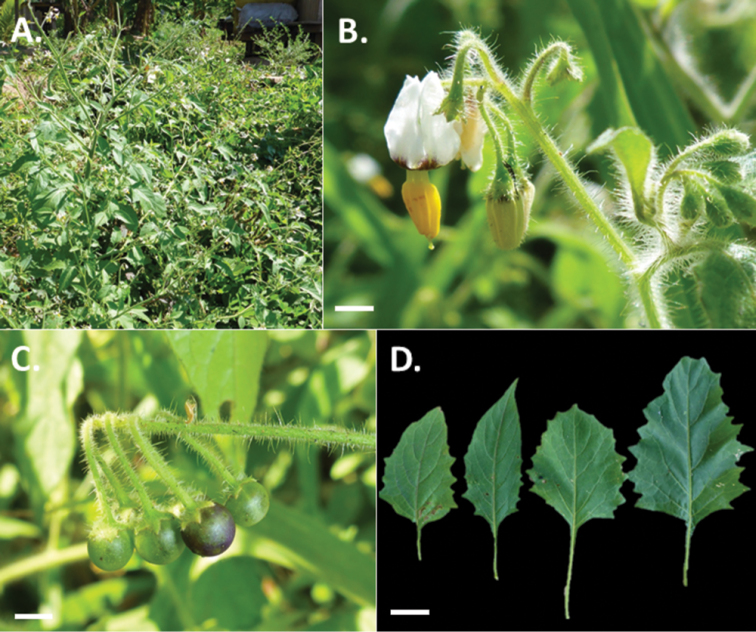
Photos of *Solanum arenicola*. **A** Habit **B** Buds and flowers, showing the dense indumentum of glandular-tipped, multi-cellular hairs throughout **C** Maturing fruits, showing reflexed pedicels in infrutescence **D** Leaf size and shape variation present within individuals as observed in the field (**A–D** *Särkinen & Balarezo 4866*). Scale bars = 1 mm. All photos by T. Särkinen.

##### Distribution.

In lowland Bolivia and Peru; in lowland moist rain forest in sandbanks and river margins, tree fall gaps, and in disturbed sites near housing and fields in open, sandy soil, with occasional records from seasonally dry semi-deciduous forests with *Hura crepitans* L. (Euphorbiaceae), *Gallesia integrifolia* (Spreng.) Harms (Phytolaccaceae), *Bougainvillea modesta* Heimer (Nyctaginaceae), and *Anadenanthera colubrina* (Vell.) Brenan (Amaranthaceae); most commonly associated with lowland rain forest pioneer species, including *Salix humboldtiana* Willd. (Salicaceae), *Tessaria integrifolia* Ruiz & Pav. (Asteraceae), *Cecropia* spp. (Urticaceae), *Calliandra* sp. (Fabaceae), *Neea* spp. (Nyctaginaceae), *Garcinia* spp. (Clusiaceae), and *Jacaratia digitata* (Poepp. & Endl.) Solms (Caricaceae), and annual herbs such as *Glinus radiatus* (Ruiz & Pav.) Rohrb. (Molluginaceae), *Physalis angulata* L., *Physalis peruviana* L., and *Solanum americanum* Mill. (Solanceae); 0–600 (1,300) m elevation.

**Figure 6.**
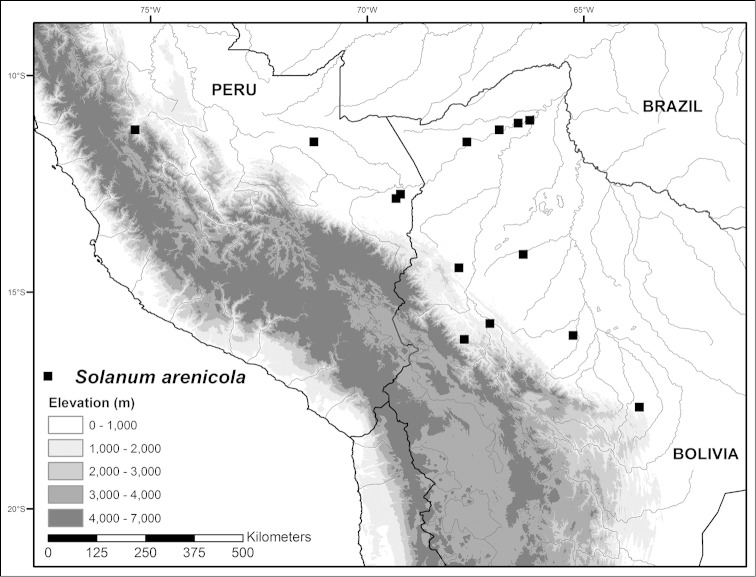
Distribution map of *Solanum arenicola* in lowlands of central and southern Peru, and northern Bolivia.

##### Ecology.

Flowering January–February and August–October, fruiting September–October.

##### Etymology.

*Solanum arenicola* is named for its habitat preference as deduced from both field observations and specimen labels. The species prefers growing on sand (*cola* = “liver on”, and *arena* = “sand”) and is most commonly collected from sand bars, loose sandy soils on land slides, at the base of tree falls on open, loosened sandy soil, or in open-sandy soils in disturbed areas around houses and cultivated plots.

##### Conservation status.

The preliminary IUCN (2010) conservation status of *Solanum arenicola* is here considered of least concern (LC) based on the relatively large EOO (c. 412,600 km^2^), although the small AOO (56 km^2^) would merit listing with endangered (EN) status. The species grows in disturbed sites along rivers, tree falls, and cultivations where bare sandy soils are available, and its association with other pioneer species indicates that the species is not sensitive to human disturbance from expanding construction and agriculture.

##### Specimens examined.

**BOLIVIA. Beni:** Prov. Ballivian, Carmen Florida, Rio Beni, 7 km upstream from Rurrenabaque, 320m, 13 Sep 1989, *D.E. Williams 960* (US); **La Paz:** Prov. Abel Iturralde, Parque Nacional Madidi, laguna Chalalan, Río Yariapo, c. 14°26'41"S, 67°53'05"W, 275m, 26 Sep 2006, *A. Araújo-M et al. 3130* (NY); Prov. Franz Tamayo, Tuichi, Río San Juan, Buenahora., 14°12'01"S, 68°39'21"W, 840m, 1 Oct 2005, *A. Araújo-M et al. 2071* (BM); **Pando:** Prov. Manuripi, large sandbar in Río Madre de Dios, 8 km (by air) NNE of Nueva Etea, 11°15'S, 66°57'W, 125m, 22 Aug 1985, *M. Nee 31497* (K); Prov. Madre de Dios, Puerto Candelaria, along the Rio Madre de Dios, 21 km (by air) WSW of Riberalta, c. 11°02'00"S, 66°15'00"W, 125m, 18 Aug 1985, *M. Nee 31398* (NY); Prov. Manuripi, Conquista, Embarcadero sobre el Madre de Dios, 150m, 2 Feb 1983, *J. Fernández Casas 8591* (NY); Along Rio Madre de Dios between Trinidadcito and San Miguel, 50 km (by air) WSW of Riberalta, c. 11°06'S, 66°31'W, 125m, 21 Aug 1985, *M. Nee 31483* (NY); Along Rio Madre de Dios, Camacho, 11°32'S, 67°42'W, 135m, 2 Sep 1985, *M. Nee 31724* (NY); **Santa Cruz:** Ichilo: Parque Nacional Amboró, along Rio Saguayo, 0.5 km NE of entrance into first Andean foothills, 1465m, 22 Dec 1988, *M. Nee 37350* (MO, US); **PERU. Cusco:** Prov. Paucartambo, 6 Aug 1974, *R.B. Foster et al. 3021* (USM); **Junín:** Chanchamayo: La Merced, 610m, 10 Aug 1923, *J.F. Macbride, 5314* (F); Chanchamayo: Rd from San Ramon past Vitoc to Union Mantish, past Rio Chilpes, 11°15'08"S, 75°21'36"W, 1309m, 2 Jun 2013, *T. Särkinen et al. 4831* (USM); **Madre de Dios:** Prov. Tambopata, Explorer'S, Inn, nr the confluence of Rio Tambopata and Rio La Torre, 39 km SW of Puerto Maldonado, 400m along the Laguna Chica Trail, c. 12°50'S, 69°20'W, 17 Jan 1989, *S.F. Smith et al. 1354* (NY).

##### Discussion.

*Solanum arenicola* can be easily distinguished from *Solanum americanum* Mill., the only other similar Morelloid species found in lowland Amazonia; it has larger anthers which are 3.0–3.2 mm long in *Solanum arenicola* as compared to the minute anthers < 1.5 mm of *Solanum americanum*. Specimens without locality information can be easily confused with *Solanum nigrescens* M.Martens & Galeotti of Central and northern South America, *Solanum aloysiifolium* Dunal of middle to high elevation Argentina and Bolivia or *Solanum subtusviolaceum* Bitter of low to middle elevation Peru and Bolivia. Both *Solanum arenicola* and *Solanum nigrescens* have simple inflorescences, but *Solanum arenicola* differs in having longer anthers (3.0–3.2 mm) compared to *Solanum nigrescens* (1.5–2.2 mm) and in the possession of glandular hairs (*Solanum nigrescens* is eglandular). The anthers are similar in size and shape to those of *Solanum aloysiifolium*, but *Solanum arenicola* has simple inflorescences and glandular-tipped hairs, while *Solanum aloysiifolium* has branched inflorescences (sometimes many branched) and lacks glandular hairs. *Solanum subtusviolaceum* possesses the same dense, glandular-haired indumentum as *Solanum arenicola*, but differs from it in having inflorescences with the flowers clustered near the tips positioned opposite the leaves rather than arising along the internodes, longer calyx lobes (2–3 mm versus 0.2–0.5 mm), more rotate corollas lobed only halfway to the base, and less exerted styles (to a maximum of 1 mm versus exerted to 3 mm beyond the anther cone).

*Solanum arenicola* is one of the few morelloids known from lowland humid forests in South America. Most morelloid species grow > 2,000 m or in drier habitats along the western slope of the Andes or in the inter-Andean valleys, whilst *Solanum arenicola* is restricted to humid forests on the eastern side of the Andes below 1,200 m elevation. Currently, the species is known from central and southern Peru and from Bolivia, but it is likely that the species also occurs in adjacent areas of Brazil in the state of Rondônia, where the Río Madre de Dios and Río Beni join and cross the Brazilian border, especially considering habitat preferences of *Solanum arenicola* for disturbed, sandy soils along river banks.

### The Potato clade

Within the non-spiny taxa of *Solanum*, the Potato clade forms a strongly supported monophyletic group (Särkinen et al. 2013) that comprises the potatoes, tomatoes and a series of smaller groups (e.g., section *Pteroidea* Dunal, Knapp and Helgason 1998, Tepe et al. 2010 and section *Herpystichum* Bitter, Tepe et al. 2011). Solanum sect. Basarthrum (Bitter) Bitter is one of these smaller groups and is distinguished by the presence of distinctive uniseriate few-celled trichomes termed bayonet hairs (sensu Seithe and Anderson 1982) and basal pedicel articulation. The taxonomy and reproductive biology of these species have been treated by Anderson and colleagues (Anderson 1975, 1976, 1977, 1979; Anderson et al. 1991; Seithe and Anderson 1982), but new species continue to be discovered in the Andes, a centre of species diversity in the group (Anderson et al. 2006; Prohens et al. 2006). The new species described here is clearly a member of this group, but has unusual calyx morphology and growth form.

#### Solanum mariae

Taxon classificationPlantaeSolanalesSolanaceae

Särkinen & S.Knapp sp. nov.

urn:lsid:ipni.org:names:77144536-1

Figs 7 –8

##### Diagnosis.

Like *Solanum caripense* Dunal, but differing in having distinctive calyx lobes 2.0–2.5 mm long and 1.8–2.0 mm wide, broadly ovate in shape and spreading in buds and flowers, and in having fruits fully enclosed in an accrescent calyx.

##### Type.

**Peru. Cajamarca:** Prov. San Marcos, Dist. Chancay, 14 km from San Marcos, just S of Chancay, on road from San Marcos to Cajabamba, 7°24'20"S, 78°07'05"W, 2606 m, 9 May 2013 (fl,fr), *S. Knapp, T. Särkinen, H.M. Baden, P. Gonzáles & E. Perales 10571* (holotype: USM; isotypes: BM [BM001034677], CPUN, E [E00700640], HUT, MOL).

##### Description.

Trailing herbs, stems to 20–30 cm tall arising from woody trailing stems that root at nodes, the individual stems up to 5 m long. Stems terete, 1.5–2.5 mm in diameter, moderately to densely pubescent with spreading bayonet hairs (uniseriate, 2-celled hairs with an elongate, thicker-walled basal cell capped by a short acuminate cell) and with simple, 2–4-celled uniseriate glandular-tipped finger hairs c. 0.5 mm long; new growth densely pubescent with trichomes like those of the stems; bark of older stems grey-brown, smooth. Sympodial units plurifoliate, not geminate. Leaves simple, 1.4–3.5 cm long, 1.0–1.6 cm wide, ovate-lanceolate; adaxial surface moderately pubescent with bayonet hairs like those on the stems, and with simple, 2-celled uniseriate glandular-tipped hairs c. 0.3 mm long; abaxial surface more densely pubescent with trichomes like those of the upper surface; primary veins 4–6 pairs; base acute to obtuse; margins entire; apex rounded; petiole 0.5–1.2 cm long, moderately to densely pubescent with trichomes like those of the stems. Pseudostipules in pairs, simple, 5 mm long, 3 mm wide, ovate-lanceolate, tip acute, resembling leaves in shape and appearance. Inflorescences 1.5–2.7 cm long, lateral and internodal, simple, with 3–5 flowers in the distal half, moderately to densely pubescent with spreading trichomes like those of the stems; peduncle 0.4–1.6 cm long; pedicels 0.6–0.7 cm long, ca. 0.3 mm in diameter at the base and apex, straight, curved at the tip, articulated at the base; pedicel scars spaced ca. 1 mm apart. Buds globose, the corolla only exerted from the calyx tube just before anthesis. Flowers 5-merous, all perfect; calyx tube ca. 1.5–2.0 mm long, the lobes 2.0–2.5 mm long, 1.8–2.0 mm wide, broadly deltate, with acute apices, spreading in bud and flower, moderately to densely pubescent; corolla 1.2–1.5 cm in diameter, shallowly stellate, white, lobed halfway to slightly less than halfway to the base, the lobes ca. 4–5 mm long and 4–5 mm wide, spreading at anthesis, moderately to densely pubescent abaxially with trichomes like those of the stem, glabrous adaxially; filament tube minute, glabrous; free portion of the filaments ca. 1.0–1.2 mm long, glabrous; anthers 2.7–3.0 mm long, ca. 2.5 mm wide, ellipsoid, yellow, poricidal at the tips, the pores lengthening to slits with age; ovary conical, glabrous; style 5–6 mm long, exerted 1.5–2.0 mm beyond the anther cone, glabrous; stigma clavate, minutely papillate, yellow-green in live plants. Fruit (immature) an ellipsoid berry, 8–9 mm long and 6.8 mm wide when developing, with the mesocarp ca. 0.2 mm wide, green, fully enclosed in the accrescent calyx, glabrous, mature fruits not seen; fruiting peduncle 1.3–2.2 cm long; fruiting pedicels 1.8–2.3 cm long, 0.3–0.5 mm in diameter at the base and 0.5–1.8 mm at apex, reflexed 180° in fruit; fruiting calyx 8–9 mm long, 3.5–4.0 mm wide and still developing, appressed to and enclosing the entire berry, the calyx lobes spreading at the mouth of enclosing tube. Seeds 30–40 per berry, 1.2–1.5 mm long, 1.0–1.2 mm wide, flattened-reniform, yellowish, the surfaces minutely pitted, the hilum positioned laterally in the middle, the testal cells pentagonal in outline.

**Figure 7.**
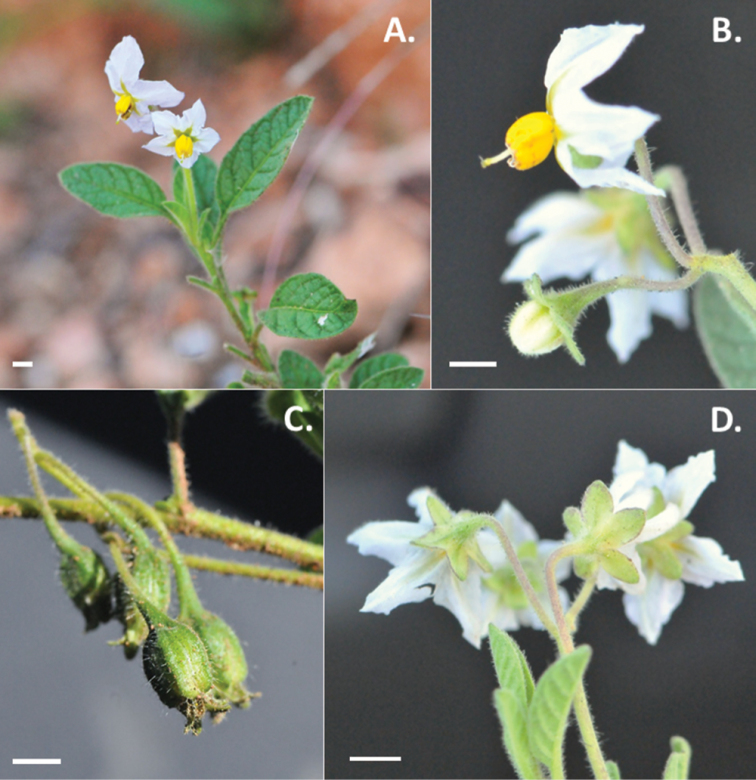
Photos of *Solanum mariae*. **A** General habit **B** A floral bud and a flower at full anthesis **C** Maturing fruits enclosed in calyx **D** Distinct calyx lobes spreading in flower (**A–D** *Särkinen & Baden 4651*). Scale bars = 2 mm. All photos by T. Särkinen.

##### Distribution.

Endemic to Peru; growing along north facing banks in loamy soils in along roadsides, not in full sun, associated with *Lycianthes lycioides* (L.) Hassl. (Solanaceae) and various grasses; only known from a single population at 2,600 m elevation from San Marcos Province in the Department of Cajamarca.

**Figure 8.**
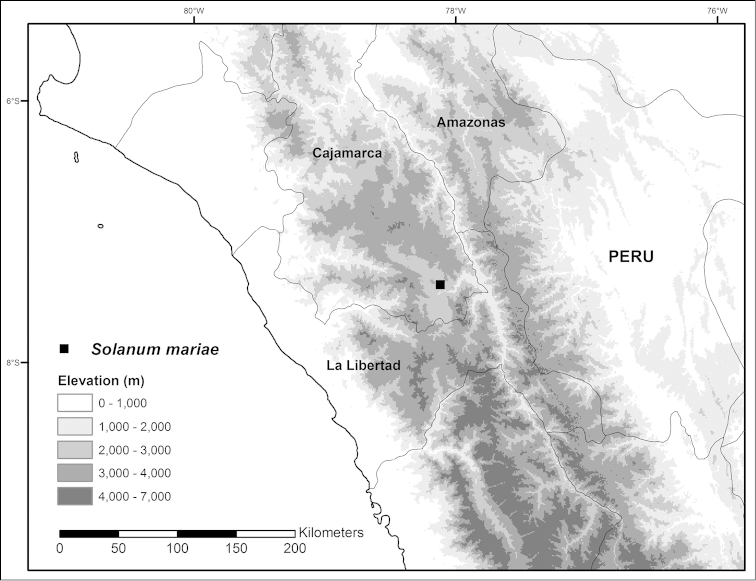
Distribution map of *Solanum mariae*, a narrow endemic from northern Peru.

##### Ecology.

Flowering and fruiting April–May.

##### Etymology.

The species is named after biologist Maria Baden who collected the first specimen in Cajamarca in 2013 with the authors. Many of the collections made in our field trips in Peru since 2006 would not have been made without her, and to honour the number of hours spend on plant spotting from fast moving vehicles and driving carefully through recent landslides, we name this shy and stunningly beautiful *Solanum* species in her honour.

##### Conservation status.

The IUCN (2010) threat status of *Solanum mariae* is here considered of critically endangered (CR) based on only a few known occurrence points near Chancay in the San Marcos province, Department of Cajamarca, Peru, with AOO of 4 km^2^. The species appears to have specialist habitat requirements, preferring north-facing shady cliff sides. The known populations are both small, and vulnerable to grazing pressures.

##### Specimens examined.

**PERU. Cajamarca:** Prov. San Marcos, c. 14 km from San Marcos towards Cajabamba, 7°24'20"S, 78°07'05"W, 2604m, 25 Apr 2013, *T. Särkinen & H.* M. Baden 46*51* (USM).

##### Discussion.

*Solanum mariae* can be easily distinguished from other section *Basarthrum* species based on the large, broadly ovate and spreading calyx lobes in flower that become to enclose the entire fruit that are not known from any other species of the section. Further distinguishing characters include the combination of trailing stems that root along nodes, the relatively dense pubescence of long, 2–4-cellular glandular-tipped finger hairs throughout the plant, and the strictly simple leaves. In Peru, the species is most similar to *Solanum caripense* Dunal and closely allied species, but differs in having simple leaves combined with the glandular-tipped finger hairs throughout mature plants and larger calyx lobes that are spreading in flower and are accrescent and enclose the fruit. Anderson and Bernardello (1991) provide a key to the members of series *Caripensia* Correll to which the new species clearly belongs based on morphology.

Based on the style extension well beyond anthers in the newly described species, *Solanum mariae* is likely to be self-incompatible. Style extension has been found to indicate self-incompatibility in sect. *Basarthrum* in previous studies (e.g., Anderson 1979).

The trailing growth form of the new species, where roots are formed at leaf nodes, has been observed in other members of sect. *Basarthrum*, as well as in the closely related sect. *Anarrhichomenum* Bitter (Tepe et al. 2012), whose members are distinguished from those of sect. *Basarthrum* by the presence of pseudostipules, winged seeds, and fruits maturing red or orange.

The central Andes region is a centre of diversity for Solanum sect. Basarthrum (Stern et al. 2008; Anderson 1979; Anderson et al. 2006; Särkinen et al. in prep.), and the new species described here adds to the growing list. *Solanum mariae* remains poorly understood based on our two collections from the single known population from the San Marcos Province of the Department of Cajamarca. No further specimens have been seen in local herbaria in Trujillo and Cajamarca. The locality lies within the watershed of the Río Marañón, and further field work in the same watershed in the Province of San Marcos is a priority in order to increase knowledge of this rare species, including the morphology of the fully mature fruits.

## Supplementary Material

XML Treatment for Solanum longifilamentum

XML Treatment for Solanum antisuyo

XML Treatment for Solanum arenicola

XML Treatment for Solanum mariae

## Supplementary materials

Supplementary material 1 Occurrence records of the four new Solanum species Data type: occurence This dataset is made available under the Open Database License (http://opendatacommons.org/licenses/odbl/1.0/). The Open Database License (ODbL) is a license agreement intended to allow users to freely share, modify, and use this Dataset while maintaining this same freedom for others, provided that the original source and author(s) are credited.Tiina Särkinen, Paúl Gonzáles, Sandra Knapp
